# Interaction of the Nanoparticles and Plants in Selective Growth Stages—Usual Effects and Resulting Impact on Usage Perspectives

**DOI:** 10.3390/plants11182405

**Published:** 2022-09-15

**Authors:** Jan Wohlmuth, Dorota Tekielska, Jana Čechová, Miroslav Baránek

**Affiliations:** Mendeleum—Institute of Genetics, Faculty of Horticulture, Mendel University in Brno, 61300 Brno, Czech Republic

**Keywords:** nanoparticles, nanopriming, antibacterial effect, seed germination, growth promotion, stress tolerance

## Abstract

Nanotechnologies have received tremendous attention since their discovery. The current studies show a high application potential of nanoparticles for plant treatments, where the general properties of nanoparticles such as their lower concentrations for an appropriate effects, the gradual release of nanoparticle-based nutrients or their antimicrobial effect are especially useful. The presented review, after the general introduction, analyzes the mechanisms that are described so far in the uptake and movement of nanoparticles in plants. The following part evaluates the available literature on the application of nanoparticles in the selective growth stage, namely, it compares the observed effect that they have when they are applied to seeds (nanopriming), to seedlings or adult plants. Based on the research that has been carried out, it is evident that the most common beneficial effects of nanopriming are the improved parameters for seed germination, the reduced contamination by plant pathogens and the higher stress tolerance that they generate. In the case of plant treatments, the most common applications are for the purpose of generating protection against plant pathogens, but better growth and better tolerance to stresses are also frequently observed. Hypotheses explaining these observed effects were also mapped, where, e.g., the influence that they have on photosynthesis parameters is described as a frequent growth-improving factor. From the consortium of the used nanoparticles, those that were most frequently applied included the principal components that were derived from zinc, iron, copper and silver. This observation implies that the beneficial effect that nanoparticles have is not necessarily based on the nutritional supply that comes from the used metal ions, as they can induce these beneficial physiological changes in the treated cells by other means. Finally, a critical evaluation of the strengths and weaknesses of the wider use of nanoparticles in practice is presented.

## 1. Introduction

Nanotechnologies have received tremendous attention since their discovery. Nanoparticles are a basic concept and building block, and they are particles that have one or more dimensions in the range of 1-100 nm. They can consist of many elements and their compounds, which are made of, especially, carbon, metals, metal oxides and various organic substances [1,2,3,4,5]. Nanoparticle dimensions can be 0D (particles with all dimensions that are in the order of nanometers), 1D (where one of the dimensions exceeds the limit of 100 nm) 2D (planar shapes with two dimensions larger than 100 nm) and 3D crystals (where all dimensions are near the limit of 100 nm) [1,6,7,8]. The shape of the nanoparticles can be flat, spherical, cylindrical, hollow core, conical, spiral, irregular, etc., and their surface can be uniform or irregular with different variations [9]. Due to their large surface area to volume ratio, they are characterized by considerable reactivity, making them excellent to use in many areas of industry [10,11,12].

The current studies show that there is a high application potential for nanoparticles in agriculture. One very common purpose is the use of nanoparticles (NPs) as nanofertilizers in order to increase crop production. Here, the division that is proposed by Liu and Lal (2015) [13] is based on the composition of the used nanoparticles that proved to be suitable. Namely, they suggested that researchers should recognize macronutrient nanofertilizers (based on N, P, K, Mg and Ca), micronutrient nanofertilizers (based on Fe, Mn, Zn, Cu or Mo, among others), nutrient-loaded nanofertilizers (for example, nutrient-augmented nanozeolites), and plant-growth-enhancing nanomaterials (which have no nutritional role but act as growth enhancers—e.g., TiO2, carbon nanotubes and graphene oxide). An important parameter from the point of view of the practical use of nanoparticles is also their stability, where we distinguish between the relatively stable NPs such as TiO_2_, Au, graphite and fullerene-related compounds, and the relatively unstable ones such as ZnO, CuO and those that are Fe_2_O_3_-based. The instability of the given nanoparticles does not always mean that they have a negative property, when, for example, the combination of micro/macro element-based NPs and their gradual release due to them having less stability can be used in the field of precise plant fertilization. Some other reviews describe this issue in more depth [14,15].

Another important group consists of the nanoparticles that are used as nanopesticides [16,17], where the strong antimicrobial effect of many types of nanoparticles is utilized. This property is most often described for silver nanoparticles, but many others are based on titanium [Ti], zinc [Zn] [18], copper [Cu] [19], chitosan [20] and graphene oxide [GO] [6] and they are effective in this manner [5,21]. It was found that by treating a plant that was infected with a pathogen, it was possible to achieve the complete destruction of the undesired microorganism, even with low concentrations of nanoparticles [3,6,22,23]. Their antimicrobial properties are affected by the shape, size and concentration of the nanoparticles [23,24,25]. The size of the nanoparticle also affects their ability to be transported into plant tissue. Many studies have confirmed that smaller nanoparticles can easily enter into the cell and are able to use more modes of transport [26].

The principle of the effect of the nanoparticles on the destruction of the microorganism cell is also diversified. A sudden change in osmotic pressure and pH due to the dissolution of the nanomaterial may be possible [27]. Another factor may be a disruption of the cell membrane and the enzymatic functions [28]. Together with complete degradation of the cell membrane, accelerated DNA fragmentation can occur [29].

A relatively new branch of application of nanoparticles in agriculture is their use in seed treatment. A seed treatment for the purpose of utilizing their improved useful properties is generally called priming, and in the case of the use of nanoparticles we call it nanopriming. It is a very promising method for influencing the basic properties of the plants before the beginning of their development [30,31].

A very important aspect from the point of view of the mobility, reactivity, biological availability, or potential toxicity of NPs is a deeper understanding of the way they interact, where also, it is necessary to consider the chemistry of colloids playing a role. To achieve higher efficiency, it is also possible to use the photocatalytic properties of some types of nanoparticles. These include nanoparticles that have the properties of semiconductors such as TiO_2_, ZnO, SnO_2_ and CeO_2_. Both of these topics are analyzed in detail by Kolenčík et al. [32].

Despite the undeniable beneficial effects of nanoparticles in various areas of agricultural production, it should be considered that there are possible risks, such as the misuse or contamination of the environment with nanoparticle residues [33,34,35,36,37]. Regarding the regulation of nanoparticle manufacturing and usage, it is necessary to keep to the rules that are established for individual states/regions. The first regulatory level has a general character and usually considers nanoparticles to be chemicals. In the case of the EU, the European Chemical legislation REACH EC 1907/2006 was introduced in 2018 with nano-specific information requirements and new provisions for their chemical safety assessment and downstream user obligations. The second regulatory level is represented by their applications in agriculture, which falls under the rules that are established for food production and safety. In the case of the EU, it is the EFSA (European Food Safety Association), which established a scientific network for risk assessment of the use of nanotechnologies in food and feed [38]. A rather comprehensive and global review that was directly focused on this issue was published by Allan et al. (2021) [39]. An additional problem with applying nanoparticles to plants is their synthesis process which may involve the use of toxic compounds such as formaldehyde, N_2_H_4_ or NaBH_4_ [40]. The natural way to mitigate this potential phytotoxicity factor is to use “green synthesis” (by using plants, fungi and other microorganisms) [5,28,41]. This principle can decrease the toxicity of the resulting nanoparticles, and their use for plant treatment is thus, more friendly to the environment [42].

Given the growing number of applications and publications, the purpose of this review is to critically evaluate the use of nanoparticles in different phases of plant growth, i.e., from seed to mature plant. Based on the comparison of a large number of articles, we were able to determine the usually observed effects on plants that were treated in this way, or the most commonly used nanoparticles. In the conclusion, the weaknesses and strengths of the individual approaches are discussed, including implications for their possible future use. We believe that this review should thus become an important knowledge support for plant scientists that are starting to use nanoparticles in the agricultural sciences, and that it will help them to quickly orientate their work in this field, and that it will facilitate the high-quality and meaningful planning of their experiments.

## 2. Current Knowledge about the Uptake of Nanoparticles by Plants

Nanoparticles may enter mature plants either by the roots or by the aboveground part of the plant, both by means of several natural physiological types of transport.

In fact, the aboveground part of the plant is naturally exposed to the nanoparticles in the atmosphere [43,44], and artificial exposure can occur during the spraying or direct injection of them into the leaf [45]. However, there is a cuticle on the surface of the plant, which is a natural barrier. It is a complex membrane with a number of important protective functions which are based on ensuring impermeability to most substances [37,46,47,48,49]. Two different uptake pathways are likely to occur: a polar pathway, through the leaf surface polar apertures, e.g., trichomes [50], hydathodes, necrosis spots and stomata, as well as a nonpolar pathway, through the leaf cuticle and its pores, which have a size of 0.2–2 nm [51,52]. The only proven way that has been performed is for the nanoparticles to enter the plants through the stomata [49,52,53,54]. Focused research has confirmed that the limiting dimensions of water-based nanoparticles that plants are naturally able to absorb through the stomata are less than 1.1 µm [55,56]. Further research has confirmed that the particles that were larger than 100 µm^3^ were found only when treating the leaves that has open stomata. Thus, a higher absorption through the stomata due to the application of a surfactant (wetting agent) was confirmed [57].

The applied nanoparticles may also have a problem penetrating the intercellular space. The diameter of the vessels usually ranged from 2 to 20 µm. In addition, larger vessels were also observed in a longitudinal section [58,59]. Further research has confirmed that the microstructure of hemp shiv exhibited 50 µm pores that were connected to 10 µm pores via 1 µm connecting pores [60]. Hence, only nanoparticles or nanoparticle aggregates with a diameter that was less than the pore diameter of the cell wall could have easily passed through and reached the plasma membrane [61,62].

An important aspect that is influencing the course of nanoparticle uptake is also the stability of the applied nanoparticles that were mentioned in the opening passage of this review. As shown by the study that is presented by Li et al. (2019) [63], with less stable nanoparticles such as those that are ZnO-based, it may happen that the given nanoparticle does not necessarily enter the plant, but rather a soluble form of zinc resulting from its breakdown does. This fact can be important in optimizing the composition and application of slow-release nano-fertilizers.

## 3. Movement and Accumulation of Nanoparticles in the Plant

Once the nanoparticles enter the plant, there are two ways that they can move through the tissues: an apoplastic and a symplastic transport. Apoplastic transport takes place outside the plasma membrane, through the cell walls of neighboring cells, and extracellular spaces and xylem vessels are what allow nanomaterials to reach the conductive tissues for further upward movement towards the photosynthetic parts of the plant [64,65,66,67]. The apoplastic pathway is important for radial movement in plant tissues and the central cylinder, as well as vascular bundles, in the phloem are what allow the distribution towards the tissues and organs in the roots [66,68,69,70]. It was also described that the particles outperform the epidermal and cortical cells by using apoplastic transport until they reach the endodermis. However, nanoparticles can form aggregates and accumulate in the endodermis due to the presence of Caspary strips, which act as a resistant barrier. For efficient translocation to shoots, the nanoparticles must then pass into the symplast [67,71,72].

Symplastic transport involves the movement of water and substances between the cytoplasm of the neighboring cells by using plasmodesmata and retina structures [70]. The effective translocation of the nanomaterials to the shoots is possible after their transfer to the symplast, thus, symplastic transport is considered more important for the transport of nanoparticles. Nanoparticles enter the intracellular space in several ways. It is possible for them to pass through by binding themselves to transport proteins, through ion channels, aquaporins, endocytosis or through the initiated formation of new pores, which may be larger than the originals [36,37,70,73]. The processes of the adsorption, translocation and accumulation of nanoparticles depend significantly on the type of plant but also on the physical and chemical nature of the nanoparticles themselves [36,37,45,71,74,75]. The transport of nanoparticles in a sympathetic way, i.e., across the membrane, and their accumulation results in an effect on the charge inside the cell, which subsequently changes the electrochemical potential of the membrane. Any changes in the electrochemical potential can affect the transport of other materials across the membrane. The electrochemical potential of the membrane should remain in equilibrium to maintain the turgor pressure, water and nutrient uptake and plant growth [76,77]. Among the important factors affecting accumulation were the crystallinity of the used nanoparticles [71,78,79], the hydrodynamic dimension of the used NPs [69,80], the character of the ambient soil, e.g., its “organic” or inorganic character [81] and/or the effect of zeta potentials [82]. The consequence of the high accumulation of nanoparticles by the plant is a high level of phytotoxicity. Gubbins et al. (2011) [83] consider that the phytotoxic effect is influenced by the size, shape, exposure time and concentration of the NPs. As evident in the review by Arruda et al. (2015) [84], where aspects of phytotoxicity are deeply analyzed, the range of the used NP concentrations is quite high, ranging from dozens of μg.l^−1^ up to hundreds of mg.l^−1^.

## 4. Application of Nanoparticles to Seeds

The group of methods that use process of soaking seeds in water or water solutions in order to improve their properties, especially during germination, are historically named as “priming methods”. On the basis of the character of the priming agents, these include hydro-priming, halo-priming, osmo-priming or hormonal priming. Nanopriming is a method of seed treatment by using some kind of nanoparticles [31,85,86]. According to its intended use, nanopriming can be divided into different applications in order to improve the health of the seed in terms of deterring its contamination by unwanted microorganisms, to increase its tolerance to abiotic stress factors and to improve the production properties of plants that are grown from such treated seeds.

Very short exposure durations are sufficient for the treatment in the case of pathogen infestation on the seed surface, but in the case of internal seed infection [87], it is usually necessary to incorporate the nanomaterials into the inner part of the seed. Nanoparticles can enter the seeds through aquaporins, a class of ubiquitous membrane proteins that are involved in the transport of water and many other small solutes [88,89]. In general, since the nanoparticles are usually applied to seeds in the form of a water solution, it is necessary to reduce the treatment of the seeds to the shortest possible duration to avoid the induction of undesirable germination. For these purposes, a vacuum can be used, which allows for the penetration of nanoparticles into the internal structures of the seed more effectively, and thus, also have the potential to affect any microorganisms that are also inside the seed [90,91,92].

### 4.1. Nanopriming to Reduce/eliminate Undesirable Microbial Seed Contamination

Concerning the fact that seeds play the role of an important and primal source of infection, especially in the case of seed-born groups of pathogens, there is still the need to develop new methods of pathogen elimination that are more effective than the current methods which are based usually on physical activities. One of the most promising approaches represents the treatment with nanoparticles [93]. As is evident, there are a very wide variety of nanoparticles that are completely different in terms of their chemical composition. At the same time, many of them have been shown to have an antibacterial effect. This suggests that the causes of the antimicrobial effect can be significantly different, as is documented by the comparison of the reviews that are focused on zinc nanoparticles [Zn] [94], selenium [Se] [95], copper [Cu] [96], silver [Ag] and chitosan [97]. The mode of action of the applied nanoparticles should, therefore, be taken into account when planning the method of seed treatment.

In terms of their antimicrobial activity, metal nanoparticles are generally well-known examples and are therefore broadly used in this context. For example, the antimicrobial effect of iron sulfide (FeS) nanoparticles on rice seeds (Oryza sativa L.) significantly reduced the incidence of *Fusarium verticillioides*. Moreover, the effect of nanoparticles was stronger than the commonly used fungicide Carbendazim. In addition, the beneficial effect of FeS nanoparticles on seed germination and seedling vitality was also observed [98].

Regarding the influence of zinc oxide [ZnO], titanium dioxide [TiO_2_] and silver [Ag] nanoparticles on the seed’s phytosanitary state and the provision of germination support, the tested nanoparticles were found to affect chili (*Capsicum annum* L.) seed germination and vitality without the phytotoxicity symptoms that are associated with excessive metal use. At the same time, they had a strong antimicrobial activity against the pathogens *Aspergillus flavus, Aspergillus niger, Aspergillus fumigatus* and *Colletotrichum capsici* [99].

The application of chitosan-guar nanoparticles (CGNP) to rice (*Oryza sativa* L.) seeds has revealed that the CGNP were activate and improved their germination, as well as protected against *Pyricularia grisea* and *Xanthomonas oryzae*, two main rice pathogens. The CGNP has also higher antimicrobial effects than chitosan or guar gum do, alone [100]. Another study that focused on the antimicrobial activity of Zn-chitosan nanoparticles against the pathogen *Curvularia lunata* showed that even low concentrations that were from 0.01 to 0.16 % had an inhibitory effect on the tested fungus. The encapsulation of zinc in chitosan caused its slower release and thus, a longer antifungal effect, and additionally, a source of plant nutrition for corn (*Zea mays* L.) [101].

Another example has described the treatment of pea seeds (*Pisum sativum* L.) with nanomaterials that are based on cinnamaldehyde that is encapsulated in alginate (biofertilizer). Due to the use of this complex, a synergic effect of growth-promoting (alginate) and antimicrobial effects (cinnamaldehyde) against *Pseudomonas syringae* pv. *pisi* was observed [102].

When assessing the suitability of nanopriming, it is also important to consider whether the treated seeds do not have a negative impact on the soil microflora, after sowing. From this point of view, Shah and Belorezova (2008) reported that lettuce (*Lactuca sativa* L.) seeds that were treated with silica [SiO_2_], palladium [Pd], gold [Au] and copper [Cu] nanoparticles displayed an antimicrobial effect in vitro but not *in situ*, which was explained by their adsorption by the substances in the soil complex [103].

A direct comparison of a seed treatment with nanoparticles and a conventional hot water treatment (HWT) in order to eliminate the pathogenic bacteria was performed by Pečenka et al. (2021). This study demonstrates the limited effectiveness of a conventional HWT in eliminating *Xanthomonas campestris* pv. *campestris* bacteria on artificially inoculated cabbage seeds. On the contrary, the highest concentration and treatment time with silver nanoparticles led to the complete elimination of Xcc contamination from the seeds [93].

### 4.2. Nanopriming to Increase Tolerance to Biotic/Abiotic Stress

Many chemicals that are used as priming compounds, including nanoparticles, have a proven potential for reducing of the stress impact on plants, including natural metabolites or synthetic chemicals [104,105]. One of the most serious problems in today’s agriculture is drought stress [30]. In this regard, the positive effect that nanopriming has was confirmed by using zinc oxide [ZnO] nanoparticles. To expand, it was found that their application to wheat (*Triticum aestivum* L.) seeds caused an increase in the formation of reactive oxygen species, mainly hydrogen peroxide [H_2_O_2_], thereby reducing the drought stress impact during seed germination. The further observations showed that there was a reduction in chlorophyll degradation, a protection of the photosynthetic apparatus and a beneficial effect on the activity of the antioxidant enzymes [106]. The impact of using silicon dioxide [SiO_2_] nanoparticles was evaluated very similarly, where a balancing of the production of the reactive oxygen species and the enzymatic activity was observed in the treated seeds. At the same time, the increase in water uptake was observed when the water was available [107]. A study examining the effect of copper nanoparticles on corn (*Zea mays* L.) seeds also revealed several positive effects that are associated with successful nanopriming. Their application increased their resistance to drought, in addition to encouraging increased germination and the subsequent vitality of the seedlings [105].

The nanopriming of melon seeds (*Citrullus lanatus* (Thunb.)) using Fe_2_O_3_ nanoparticles did not show a phytotoxic impact on the plants, in contrast to that caused by bulk amounts of Fe_2_O_3_ and FeCl_3_. In addition, the activation and induction of defense responses have been reported and as well as this, the levels of jasmonic acid and its precursor 12-oxo-phytodienoic acid (OPDA) were increased [108].

The application of Zn-chitosan-based nanoparticles to corn (*Zea mays* L.) seeds led to an increase in their resistance against the fungus *Curvularia lunata*. The antimicrobial properties of the nanoparticles themselves were supported by the increased activity of the defense enzymes within the nanopriming-derived plants [101].

Regarding the stress that is due to contaminated soil, research that has been performed from this point of view has shown that nanoprimed seeds can reduce the uptake of contaminants, thereby reducing their phytotoxicity and the negative impact that they have on plant growth and health. For example, the effects of manganese [109] nanoparticles that were applied to pepper seeds (*Capsicum anuum* L.) which were grown in an environment with increased salinity showed a significantly lower concentration of salt ions inside the plants [110]. The problem of increased salinity in the substrate was also solved in the research, where iron oxide [Fe_2_O_3_] nanoparticles were used at various concentrations on sorghum (*Sorghum bicolor* (L.) Moench) seeds, both during seed activation and as a preventive treatment against increased salinity. The effect of the soil salinity caused a reduction in the chlorophyll content, photosynthesis rate and transpiration, increased the lipid peroxidation, and decreased their growth. The use of nanoparticles improved the germination process and subsequent seedling growth, along with eliminating the effects of the stress caused by increased environmental salinity [111]. The reduction in these stress impact effects was also tested using zinc oxide [ZnO] nanoparticles. Untreated lupine plants (*Lupinus albus* L.) showed stress symptoms, decreased growth, and a lower production of photosynthetic pigments. On the contrary, plants that were grown from the seeds that were treated with ZnO nanoparticles did not exhibit these stress symptoms and showed a lower sodium uptake [112]. Another study compared the hormonal balance in the germination of rapeseed (*Brassica napus* L.) that was stimulated by the stress from the saline environment and after a treatment with selenium [Se] and zinc oxide [ZnO] nanoparticles. It was reported that the nanoparticles cause evident physiological phenomena that minimized the stress from the salinity of the environment [113].

The reduction of the stress impact from a substrate that was contaminated with cadmium [Cd] was achieved, also, by the treatment of wheat seeds (*Triticum aestivum* L.) with zinc oxide [ZnO] and iron [Fe] nanoparticles. There was an increase in the concentration of Zn and Fe in the roots, and thus, a decrease in Cd uptake was observed. Moreover, the tested plants showed an increased biomass production due to the improved physiological conditions and reduced Cd accumulation [114]. In a similarly conducted study, with the use of silicon [Si] nanoparticles, comparable results regarding the cadmium content reduction and biomass increase were achieved [115]. Possible reasons why the treatment with nanoparticles can affect other ion distributions are discussed in Section 5.2.

The physiological effects of iron [Fe] and copper [Cu] nanoparticles on wheat (*Triticum aestivum* L.) seeds were observed in the study by Yasmeen et al. (2017), where the application of 25 ppm of both nanoparticles activated between 25 and 121 proteins that are involved in the process of seed germination. As a consequence, the increased production of other substances that are responsible for the activation of proteins and enzymes was found. As a result, significant physiological support for the association of seed germination and resistance to stress factors has been achieved [116].

From the point of view of the internal processes in the nanoprimed seeds, nanoparticles have been shown to affect phytohormone production, such as the production of abscisic acid (ABA), gibberellic acid (GA) and others. In summary, the presence of nanoparticles in the seed tests, through several chains of subsequent events, induces the accumulation of reactive oxygen species that are a common part of the processes that are associated with the end of dormancy [117,118,119]. Their presence and absence can also significantly increase the content and production of the phytohormones that are involved in seedling development and plant defense responses [101,108].

### 4.3. Seed Nanopriming to Improve Plant Performance

Most of the nanoparticles that are used for seed nanopriming have, among other properties, a significant effect on improving seed germination and the plant’s vitality. The increase in the rate of germination is of great importance, especially for plants that, for various reasons, show a naturally reduced rate of germination. A typical example are this are the triploid watermelon varieties (*Citrullus lanatus* (Thunb.)), which are valuable especially for their seedless fruits. The mechanisms of silver [Ag] nanoparticles effects, in combination with those of turmeric oil nanoemulsions (TNE), were studied after their application to cultivars ‘Riverside’ (diploid) and ‘Maxima’ (triploid) seeds. The nanoparticle application contributed to an increase in the rate of germination, metabolic activity, an increase in biomass production and therefore, a higher yield [120].

As is evident, silver [Ag] nanoparticles have a proven antimicrobial effect, but the effect of increased seed vitality after their application was also observed. As an example, are the results of a study on the brief exposure of aged rice seeds (*Oryza sativa* L.) to silver nanoparticles that were formed by green synthesis and their subsequent drying. This measure caused an increase in the enzyme activity and as a result, the rate of germination and the subsequent seedlings’ development were also increased [85]. The beneficial effect of silver [Ag] nanoparticles has also been observed when they were applied to aged bean (*Vicia faba* L.) seeds. The effect of these nanoparticles was manifested as a reduction of the genotoxic effects that are associated with seed aging [121].

Advanced magnesium oxide [MgO] nanoparticles were used for a mung (*Vigna radiata* L.) seed treatment, where their germination was increased, and the elongation of the seedlings was promoted [122]. Mung seeds were used also by Sarkar et al. (2021) for their treatment with copper oxide [CuO] nanoparticles that were coated with APTEX (3-Aminopropyl) triethoxysilane)) which was synthetized using coriander extract (*Coriandrum sativum* L.). They observed a similar positive effect on germination and increased their water uptake, as was true in the case of the MgO NPs [123]. Manganese [109] nanoparticle nanopriming has also been investigated due to the possible effect that it has on nitrogen uptake and the metabolism of mungo seeds (*Vigna radiata* L.) [124].

The application of platinum [Pt] nanoparticles that were stabilized with polyvinylpyrrolidone to pea (*Pisum sativum* L.) seeds resulted in the inhibition of rhizobial colonization and arbuscular mycorrhizal fungi. Despite that negative effect, the treated plants produced more seeds in shorter period of time in comparison to the control plants, nevertheless, the seeds had a lower weight. This effect can also be considered as the nanopriming of seeds as it increased the plant’s productivity [125].

Research on the effect of metal sulfide nanoparticles, namely silver sulfide [Ag_2_S] and zinc sulfide [ZnS], in promoting the seed germination of soybean (Glycine max L.) and wheat (*Triticum aestivum* L.) has shown a positive effect on the seeds’ germination rate and growth parameters [126]. Other results have confirmed that the nanopriming of corn seeds (*Zea mays* L.) with Zinc [Zn]-encapsulated chitosan nanoparticles has a positive effect on seed germination. Simultaneously, zinc [Zn] deficiency in plants was compensated, and an antimicrobial effect against the pathogenic fungus *Curvularia lunata* was observed [101].

In order to increase the germination of rice (*Oryza sativa* L.) seeds and the vitality of the seedlings, Afzal et al. (2021) used two forms of iron particles: iron oxide [FeO] that was phytochemically stabilized by an extract from the flowers of *Cassia occidentalis* L., and iron sulfate [FeSO_4_]. In the results, the FeO nanoparticles showed a more favorable effect on plant growth when it was compared to that of FeSO_4_ [127]. The nanopriming of wheat (*Triticum aestivum* L.) seeds with iron oxide nanoparticles [Fe_2_O_3_] to increase seed germination was described in another study. Interestingly, the treatment of the seeds caused an increase in iron [Fe] biofortification and accumulation in the produced seeds [128]. Another Fe-based nanoparticle was used as a stabilizer on a silica substrate and applied to the soil. Subsequently, maize (*Zea mays* ‘Single 704’) and barley (*Hordeum vulgare* ‘Valfajr’) seeds were sown to this substrate. The shortening of the germination time, an increase in the growth rate and biomass formation were observed [129].

**Figure 1:**
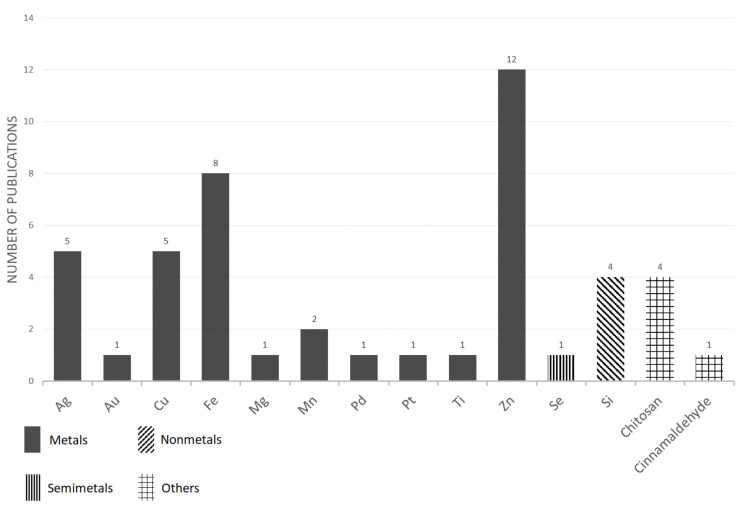
The number of papers reporting various nanoparticles as found for seed treatment—an overview.

However, a clear positive effect of nanoparticle treatment was not always observed. For example, nanopriming which was performed on wheat (*Triticum aestivum* L.) seeds with the use of zinc oxide [ZnO] nanoparticles confirmed that it has a beneficial influence on seed germination, but it negatively affected the subsequent development of the plants [130]. This observation was confirmed in another study, where four types of Zn-based nanoparticles did not have a negative effect on the germination rate, however, an inhibitory effect on the root and the shoot elongation of Chinese cabbage (*Brassica pekinensis* L.) plants were observed [131]. The selected information from the articles that used nanoparticles for seed treatment is summarized in Table 1 and Figure 1. As is evident, the most popular types of nanoparticles that were used for seed treatments were those that were based on Zn, Fe, Cu and Ag. The possible reasons for this distribution are discussed in Section 5.2

## 5. Application of Nanoparticles to Plants

The targeting, using the nanoparticle-based substances, certain areas of plant seedlings or adult plants often depends on what the intended effect on the plant is. Most frequently, it is the foliar application, i.e., spraying on the leaf area, but examples of applications on other target areas include on the root part, the substrate (hydroponics), or on the flowers, which have also been presented. The vast majority of the available literature is focused on the applications for preventive or curative interventions against plant pathogens or the harmful effects that they have, and also as fertilizers. The following two chapters will be devoted to these two areas, in particular. Nevertheless, both of these effects can overlap, where those plants with good fitness express a higher tolerance for biotic or abiotic stressors [17,134,135,136].

### 5.1. Treatment of Plants with Nanoparticles for Protection against Pathogens

Regarding antimicrobial, fungicidal and antiviral properties, the metal elements and ions have been generally reported as very usable in protecting plants against pathogens (see Table 2), and factually, silver [Ag]- and copper [Cu]-based nanoparticles have frequently shown a significant inhibitory effect against a wide range of pathogens [4,5,137,138]. For example, silver nanoparticles [Ag] that are biosynthesized by using stem extracts from cotton plants (*Gossypium hirsutum* L.) were tested for their protection capabilities against *Xanthomonas axonopodis* pv*. Malvacearum* and *Xanthomonas campestris* pv*. Campestris* on cowpea plants (*Vigna unguiculata* L.). The antimicrobial effects against both pathogens have been observed and simultaneously, any phytotoxicity of the used nanoparticles has been registered [109]. By applying silver [Ag]-chitosan nanoparticles to strawberry fruits (*Fragaria* × *ananassa* Duchesne ex Rozier), a significant reduction in the incidence of *Botritis cinerea* was achieved [139]. The nanosized silver–silica [Ag-SiO_2_] hybrid complex was applied to the *Arabidopsis thaliana* L. plants, where an increased expression in systemic acquired resistance (SAR) marker genes was observed and simultaneously, a significant increase in their resistance to *Pseudomonas syringae* pv. *tomato* was achieved [140]. To protect tomato plants (*Lycopersicon esculentum* Mill.) against *Xanthomonas perforans*, the plants were treated with special DNA-directed silver [Ag] nanoparticles which were grown on graphene oxide (GO). Their inhibitory effect on the bacterium *X. perforans* was observed under in vitro conditions and the treated plants also showed a significantly lower severity of the symptoms [141].

Copper particles have also very wide spectra of applications, including for antimicrobial purposes, especially when they are used in an acidic environment. Copper substances that do not meet the parameters of the nanoparticles are frequently used in plant protection, whereas a very high percentage of total Cu consumption in agriculture represents the protection of vineyards against fungi of the genus *Perenospora,* but also *Botriotinia* [142,143,144,145]. Assuming that there is a significantly higher efficiency of copper nanoparticles when they are compared to the conventional preparations, it is thus possible to expect significantly less environmental contamination by using nanoparticle-based materials.

After penetrating the intracellular space, copper interacts with the organelles, forming reactive oxygen species that induce a number of important biochemical reactions, especially the degradation of lipids and proteins. The result can be cytotoxicity, the disruption of cellular functions and DNA damage [26,146], which are effects that are desired in the case of antimicrobial applications. The soil application of biosynthesized copper [Cu] nanoparticles has caused a reduction in the incidence of red root-rot disease (pathogen *Poria hypolateritia*) in planted tea plants (*Camellia sinensis* Kuntze). A trivial variation of microorganisms in the soil, and an increase in the leaves that were harvested from the plants were observed [147]. Several forms of copper [Cu]-based nanoparticles, namely CuO, Cu_2_O and Cu/Cu_2_ nanocomposite, have been studied by Giannousi et al. (2013). They were applied to tomato plants (*Lycopersicon esculentum* Mill.) in order to protect against the *Phytophthora infestans* pathogen. The results have showed that the nanoparticles that were used were effective and they need lower effective amounts than commercial agrochemicals do [148]. Copper-chitosan nanoparticles were applied also to millet (*Eleusine coracana* Gaertn.). Seed nanopriming has caused growth promotion. Subsequently, the leaf treatment by these nanoparticles and the inoculation by the pathogen *Pyricularia grisea* were performed. The application of the nanoparticles caused a significant increase in the defense enzyme production, and the treated plants showed a lower degree of infestation [149].

However, there are also articles where the use of Ag or Cu nanoparticles have not shown unambiguous beneficial effects. For example, the research of antioxidant enzymes that are accompanied with the stress response has shown that treating the leaf area of wheat seedlings (*Triticum aestivum* L.) with silver [Ag] nanoparticles against the pathogen *Fusarium culmorum* can have negative phytotoxic effects, which are comparable to the damage caused by *Fusarium* [135]. In another study, the application of silver [Ag] and copper [Cu] nanoparticles to summer oak (*Quercus robur* L.) seedlings did not cause a reduction in the occurrence of oak powdery mildew (*Erysiphe alphitoides*). Also, no effect on seedling growth was observed, but quite surprisingly the increased colonization of the roots by ectomycorrhizal fungi was observed in the treated plants [138].

Regarding another element, the effects of zinc [Zn] nanoparticles were also frequently evaluated as being excellent when they were used against several pathogens. For example, He et al. (2011) reported about the activity against *Botrytis cinerea* and *Penicillium expansum* [150]. Wani and Shah, (2012) confirmed the activity of Zn-based nanoparticles against *Fusarium oxysporum* and *Mucor plumbeus*, and similarly, Sardella et al. (2017) reported the same findings against *Alternaria alternata* and *Rhizopus stolonifer* [151]. More specifically, the application of the nanomaterials that are based on zinc oxide [ZnO] and named as Zinkicide SG4 and Zinkicide SG6, were evaluated in terms of their antagonism against *Xanthomonas citri* subsp. *citri*. The infested plants were grapefruit trees (*Citrus* × *paradisi* Macfad.), and the observed protective effect that they had was better when they was compared to the copper compounds that are used in commercial pesticides [151,152]. Simultaneously, their efficacy against *Escherichia coli* and *Xanthomonas alfalfae* subsp. *citrumelonis* has also been observed [153]. The significant inhibition of *Botritis cinerea* was achieved by applying zinc oxide [ZnO] nanoparticles and photoactivated zinc oxide [ZnO] nanoparticles to strawberry plants (*Fragaria* × *ananassa* Duchesne ex Rozier) [154].

Carbon-based nanoparticles also show interesting antimicrobial effects against many microorganisms [155]. These compounds theoretically provide a huge range of shapes and properties, from polyatomic planar to rod-shaped, spherical or voided. This fact also affects the principle of their antibacterial effect, which is based, for example, on physical/mechanical damage, the photothermal effect, the inhibition of bacterial metabolism or oxidative stress-inducing factors. A comprehensive view of this topic is presented by Xin et al. (137). The main uses of carbon-based nanoparticles in phytopathology are represented, namely, by forms such as fullerenes, graphene oxide and carbon nanotubes. From this point of view, the highly important results which are presented by Wang et al. (2013), where the effect of several forms of carbon nanoparticles [C] as single-walled carbon nanotubes (SWCNTs), graphene oxide (GO), multi-walled carbon nanotubes (MWCNTs), reduced graphene oxide (rGO) and fullerene (C_60_), were compared on the base of their activity against *Ralstonia solanacearum* [156]. It was found that all of the used nanomaterials disrupted the cell wall and released cytoplasmic materials from the bacterial cells. GO and rGO were used also against the bacterium *Xanthomonas oryzae pv. oryzae* by Chen et al. (2013). They observed a strong inhibitory effect on bacterial growth, but a stronger effect was observed from the use of GO [157]. The antifungal activity of six carbon nanomaterials (CNMs, single-walled carbon nanotubes (SWCNTs), multi-walled carbon nanotubes (MWCNTs), graphene oxide (GO), reduced graphene oxide (RGO), fullerene (C_60_) and activated carbon (AC)) was tested, also, against *Fusarium graminearum* and *Fusarium poae.* The results showed that, except for C _60_ and AC carbon [C], all of the tested nanoparticles have significant antifungal properties [158]. The effects of graphene oxide (GO) nanoparticles and zinc oxide [ZnO] nanoparticles have also been tested on carrot (*Daucus carota* L.) plants for their effect on the pathogens *Pectobacterium carotovorum*, *Xanthomonas hortorum* pv. *carotae*, *Meloidogyne javanica*, *Alternaria dauci* and *Fusarium solani*. The zinc nanoparticles had a slightly stronger inhibitory effect on pathogen growth than the graphene oxide (GO) nanoparticles did [159].

Nanoparticles that are based on elemental sulfur represent another group with a confirmed antimicrobial effect. The antagonistic effects that they have on *Pseudomonas areuginosa*, *Staphylococcus areus*, *Candida albicans*, *Aspergillus flavus* [160], *Aspergillus niger*, *Fusarium oxysporum* [161], *Fusarium solani and Venturia inaequalis* [162] have been confirmed.

The use of magnesium oxide [MgO] nanoparticles on tomato roots (*Lycopersicon esculentum* Mill.) against *Ralstonia solanacearum* inhibited pathogen growth [163]. Alumina nanoparticles [Al_2_O_3_] were used for a tomato root treatment (*Lycopersicon esculentum* Mill.) to avoid the tomato root rot that is caused by *Fusarium oxysporium,* and they were compared with tolclophos-methyl, a substance that is used in commercial plant protection products. The efficacy of both substances was similar; the occurrence of the pathogen was significantly suppressed. However, a significant beneficial effect on plant growth properties was observed in the variant that was treated by nanoparticles [164].

Further studies point to the development and use of pesticides with controlled release [165]. The controlled release has been achieved in different ways [166], and in many of which, various nanomaterials and modifications of nanomaterials have been used [167,168,169]. The result was a controlled release of nanopesticides (a nanomaterial structure with a bound pesticide) [170,171,172,173].

Another modification of the nanomaterial application arose with the development of a method that covering the plant leaf with a layer of a disposable pesticide [174]. Yu et al. (2017), in their study, report that applied pesticides can withstand light radiation and prevent the cucumber leaf (*Cucumis sativus* L.) from attack by the pathogen. The active substance that was used was abamectin, a pesticide from the soil bacterium *Streptomyces avermitilis*, which was encapsulated in polylactic acid (PLA) nanoparticles. It is an easily biodegradable material, protects abamectin from light radiation and is easily biodegraded when the leaf is attacked by the pathogen, which occurs with release of the active substance [10].

### 5.2. Use of Nanoparticles to Increase Plant Productivity and Resistance to Stress

The rising demand for food production and profitability has forced farmers to increase their use of fertilizers. However, the supply of mineral fertilizers is declining. They also have adverse effects on the environment, due to the leaching of nutrients from the soil, or their mineralization into forms that are unacceptable for plants. A new opportunity has arisen for the use of biofertilizers, which consists of the application of living or latent cells of symbiotic microorganisms. These are most often species that are colonizing the rhizosphere (e.g., *Clostridium pasteurianum*, *Azotobacter* sp., *Azospirillum* sp., *Rhodobacter* sp., etc.) or the inside part of the plant (e.g., *Rhizobium* sp. and *Anabaena* sp) [14,190,191]. The use of nanotechnologies is an another option that can increase the efficiency of fertilizers in agriculture [192].

The treatment of coffee (*Coffea arabica* L.) plants with zinc sulfate monohydrate [ZnSO_4_ · H_2_O] or zinc oxide nanoparticles [ZnO] was compared by Rossi et al. (2019). The plants that were treated by ZnO showed a much higher content of zinc [Zn] than the other variants. A significant increase in the fresh weight and dry weight of roots and leaves was also observed [175]. The application of zinc oxide [ZnO] nanoparticles to Clusterbean plants (*Cyamopsis tetragonoloba* L.) caused an increase in their growth and biomass production, and an increase in the enzymatic activity and chlorophyll in plants, as well as an increased rhizospheric microbial population [176]. Zinc oxide [ZnO] nanoparticles and normal-sized zinc oxide [ZnO] particles were compared on the basis of chickpea plants treatments (*Cicer arietinum* L. ‘HC-1’). A significant effect on the variant that was treated with nanoparticles was observed on biomass production and enzyme activity. Moreover, the nanoparticles did not show phytotoxicity, while standard ZnO inhibited root growth [24]. ZnO-based nanoparticles were also foliar-sprayed on cereal foxtail millet [193] and lentil plants [194]. The grains originating from a treated plant of foxtail millet had significantly higher oil and total nitrogen contents when they were compared with those of the control. Both experiments had a common effect where a significantly lower crop water–stress index was observed. The effect of zinc [Zn], iron [Fe] and manganese [Mn] [109] nanoparticles on the growth properties of beans (*Phaseolus vulgaris* L.) was compared by Marzouk et al. (2019) [177]. An increase in the plant’s growth properties and an increase in its biomass production were observed in all of the treated variants, but the best results were observed for zinc [Zn]. The effect of titanium dioxide [TiO_2_] and zinc oxide [ZnO] nanoparticles of a similar size on tomato growth (*Lycopersicon esculentum* Mill.) was compared by Raliya et al. (2015). The results showed a critical concentration limit for both of the nanoparticles, up to which the plant’s growth and development are promoted, with no improvement beyond that. The better uptake of nanoparticles from foliar spraying than from the soil was also observed [178]. A comparison of TiO_2_- and ZnO-based nanoparticles was also performed on sunflowers [11]. Somewhat surprisingly, the ZnO-NP treatment induced generally better sunflower physiological responses, while the TiO_2_-NP treatment significantly affected the quantitative parameters such as early maturation and oil content.

The foliar application of silver nanoparticles [Ag] was performed on the plants *Triticum aestivum* L. ‘UP2338’, *Vigna sinensis* (L.) Endl. ex Hassk. ‘Pusa Komal’ and *Brassica juncea* L. ‘Pusa Jai Kisan’. A predominantly positive effect on their growth parameters was observed. The effect of the treatments on the bacterial diversity of soil was also investigated [179].

Chitosan-based nanofertilizers containing nitrogen [N], phosphorus [P] and potassium [K] were investigated by Abdel-Aziz et al. (2016). The chitosan-NPK nanoparticles were applied by foliar spraying, and they easily penetrated into the wheat plants (*Triticum aestivum* L.). The treated plants showed faster growth, reached harvest maturity earlier and a higher yield was observed [180].

It was also proved that the effect of graphene oxide on bacterial microflora (*Rhizobium* sp. E20-8) in soil is negligible, but there was a significant improvement in their drought stress resistance. This led to an increase in the yield of corn plants (*Zea mays* L.) [181].

Iron oxide nanoparticles [Fe_2_O_3_] and a chelated-Fe fertilizer (ethylenediaminetetraacetic acid-Fe; EDTA-Fe) were investigated by Rui et al. (2016). The nanoparticles were applied to peanuts (*Arachis hypogaea* L.), where the regulation of phytohormone contents and antioxidant enzymatic activities were observed. The nanoparticles caused an improvement in the plant’s growth and a greater biomass recovery [182]. Several forms of iron [Fe] and zinc [Zn] nanoparticles were applied to the leaves of fodder corn plants (*Zea mays* L.) by Sharifi et al. (2016). It had a significant effect on the amount of chlorophyll in the leaves, the biomass formation, its growth rate and the substance content that was observed [183]. An increase in the growth properties and a greater grain production was achieved also by the treatment of barley plants (*Hordeum vulgare* L.) with titanium dioxide [TiO_2_] nanoparticles, chelated zinc oxide [ZnO] nanoparticles and iron oxide [Fe_2_O_3_] [184].

From the current knowledge, it can be deduced that nanoparticles can also very effectively affect the uptake of ions by plant roots. It is assumed that the influencing of the transport of other ions after a nanoparticle treatment can be caused by the formation of apoplastic barriers; some nanoparticles can also bind with metallic ions and make them unavailable. Another possible strategy is that a treatment with NPs induces the production of structural protective agents or activates the oxidation defense system of a plant. This topic is reviewed by Zhou et al., (2020) [195] who place a special focus on this situation with the use of metal ions. In this regard, it has been found that titanium dioxide [TiO_2_] nanoparticles with an anatase or rutile structure can reduce the uptake of arsenic [As] that is performed by rice plants (*Oryza sativa* L.) that are grown in contaminated soil. The accumulation of arsenic in the root part, shoots and seeds was limited, with nanoparticles with an anatase structure having a slightly better effect on them [136]. Similarly, it was verified that the modified cerium dioxide [CeO_2_] nanoparticles can interact with cadmium [Cd] and arsenic [As] which results in a reduction occurrence and the uptake activities by the plant from the nutrient solution. The character of these interactions are strongly dependent on the pH, which is highly affected by the root exudates, if we are to focus on the situation in the root system [185]. In another study has focused on Cd remediation. Rizwan et al. (2019b) applied silicon [Si] and titanium dioxide [TiO_2_] nanoparticles by foliar spraying them onto rice plants (Oryza *sativa* L. ‘Kainat’) that were grown in a substrate that was contaminated with cadmium [Cd]. A lower phytotoxic effect, an increase in the biomass production and a lower accumulation of cadmium were observed in the plants, after the treatment [186]. As an explanation for this, the authors have hypothesized that the beneficial effect of the nanoparticles was mainly driven by the improved photosynthetic apparatus and by the elimination of the plant growth inhibition that is usually observed in the case of Cd ions exposition.

The research by Bao-shan et al. (2004) focused on forest nurseries and found that silica nanoparticles [SiO_2_] have stimulating effects on the growth of larch (*Larix* *olgensis* A. Henry) seedlings. At the same time, it was found that soaking the roots of the seedlings in a solution of nanoparticles which was diluted to 500 μL.l^−1^ causes a noticeable increase in the amount of chlorophyll [187]. Similar tendencies were confirmed by testing silica nanoparticles [SiO_2_] on hawthorn (*Crataegus azarolus* L.) and mahaleb (*Prunus mahaleb* L.), and that experiment was performed by Ashkavand et al. (2018). The application of nanoparticles caused a decrease in the water potential of RWC (relative water content), but there was a slightly increased growth of the above-ground and underground parts of the plants, more significantly for hawthorn. In addition, different concentrations of silica nanoparticles did not affect the concentration of the macroelements. However, at higher concentrations of the tested nanoparticles, phytotoxicity was observed [188]. In this case, it can be hypothesized that the phytotoxic effect that was observed by them could be due to the changes in pH. As presented in [196], this effect can appear at higher concentrations of this kind of nanoparticle.

An interesting group is represented by nanofertilizers with a controlled release. This approach is very promising, among other applications, for soil fertilization, where standard fertilizers are quickly washed out or inactivated for use by plants, because the bonds that are established in nanoparticles are more stable [16]. Various nanocomposites are used as adsorption matrices in a study where Jatav et al. (2013) describe this issue and emphasize that the superadsorbent polymers are promising. Among the most used synthetic polymers are polylactide and polylactide—polyglycolide copolymers, polycaprolactones and polyacrylates [197]. Of the natural polymers, alginate, albumin and chitosan have been widely studied [192]. Regarding chitosan, 78 nm nanoparticles have been used for the controlled release of NPK [198]. Preetha et al. (2017) used various nanoclays and zeolites as adsorption matrices for the release of artificially added phosphate [PO_4_] fertilizer, wherein zeolites appear to be more promising for the controlled release of phosphate [199].

A very promising aspect of nanoparticle use is that there usually exists a wide range of structural or surface modifications that affect their original properties. An example is the encapsulation of a less acceptable element by the envelope of a more easily received substance. For example, nanoparticles with a copper core and a carbon shell [C] were better absorbed by plant roots (*Taxodium distichum* L.) than they were by copper ions alone. The result was an increase in copper uptake by the plants [189]. It is also necessary to note that copper has also been found to be a nanoparticle with the ability to bioaccumulate in plant and animal bodies [26], and the increased intake of copper [Cu] nanoparticles has been even shown to cause a phytotoxic reaction, as reported by Lee et al. (2008) for the wheat (*Triticum aestivum* L.) and bean (*Phaseolus* sp.) plants that expressed a growth retardation [200].

**Figure 2:**
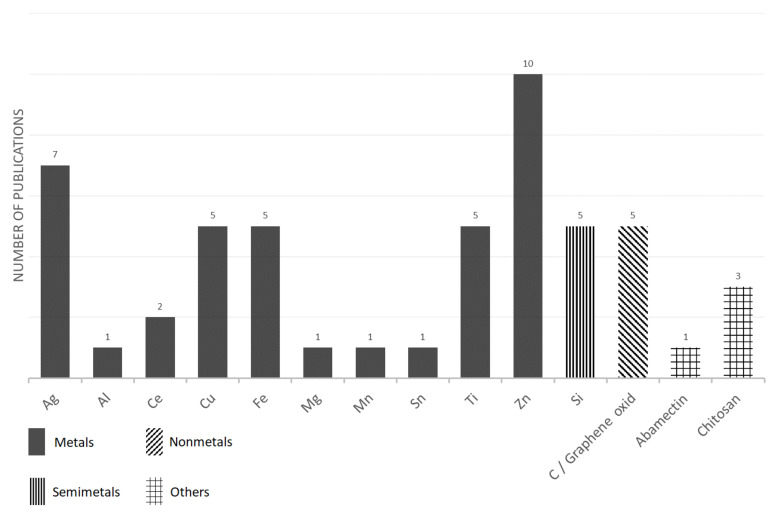
The number of papers reporting various nanoparticles as found for plant treatment—an overview.

The selected information from articles that used nanoparticles for plant treatments are summarized in Table 2 and Figure 2. In summary, the most popular types of nanoparticles that are used for seed or plant treatments are those that are based on zinc [Zn], silver [Ag], iron [Fe], copper [Cu], titanium [Ti] and silica [Si]. Several factors are likely involved in this layout of individual NP popularity. In the case of the Ag-based NP’s popularity, their historical role may play a role, as just silver-based nanoparticles were one of the first to be widely used in practice due to their relatively easy preparation [201]. It is also evident from the above list that most applications are oriented towards nanoparticles containing micro or macro elements. As a possible explanation for this trend, it is the authors’ assumption that the application of this kind of nanoparticle will induce a synergistic effect in terms of a positive impact on nutrition and at the same time, it will induce the general positive properties that have been described for nanoparticles, such as the effect on ROS production, a stress resistance, a higher photosynthetic activity, etc. On the contrary, nanoparticles with expensive inputs, such as Au-based or Pt-based NPs, are rarely used in experiments. This is despite the fact that their availability is quite wide in terms of the number of companies supplying nanoparticles, and that their application may probably also achieve an effect in terms of an antimicrobial effect or a positive effect on growth. However, their use in practice would most likely be unprofitable.

## 6. Conclusions

The use of nanomaterials already brings breakthrough discoveries across various disciplines. At the time of the beginning of the use of nanoparticles in plant production, theoretically, it was possible to recognize some usable general properties of nanoparticles such as their antimicrobial effect, the induction of the biochemical pathways that are included in the stress reaction, the need for a much smaller dose of them to achieve the desired effect or their higher stability with the possibility of the gradual release of the nutrients. The information that has been presented in this study, i.e., compared to the factual effects that have been observed in many different studies, and it has thus revealed the most reasonable areas for nanoparticle application. One such area represents nanopriming, where the improvement to seed germination, the reduced pathogen contamination of seeds and the improved stress tolerance that it generates are the most probable potential benefits. In the case of the application of nanoparticles directly on the plant, the improvements to the plant’s growth and the elimination of plant pathogens were the most frequently registered benefits. Thus, it is evident that by utilizing the nanoparticles, it is possible to achieve higher profitability in production, but also, the environmental aspect of their use is very important.

Due to the very high efficiency of nanoparticles, for example, their use would significantly reduce the consumption of agrochemicals. This environmental benefit is even more pronounced in cases where, instead of conventional agrochemicals, the NP-based alternatives would start to be used. An example is that the environmentally problematic copper-based plant protection products that are offered could be replaced with a suitable Cu-NPs alternative and this would mean there would be several times smaller contamination quantities with this heavy metal. On the environmental aspects, it is also necessary to mention the still not well described impact that nanoparticles have on the environment and human health. In this respect, for example, there is a relatively significant gap in knowledge regarding the impact of the use of nanoparticles on soil properties, when theoretically, their antimicrobial effect can negatively affect the soil microbiome.

The relatively evident beneficial effects that they have and, in contrast, an incomplete picture of their impact on the environment, also encourage fundamental questions regarding the classification of the use of nanoparticles in crop production systems. Does their use belong to the practice of precise agriculture, sustainable agriculture or something else entirely? If we recognize the environmental aspect as one of the important factors that is limiting the wider use of nanoparticles, we can emphasize, here, the existence of nanopriming, i.e., the seed treatment strategy. Here, this strategy has shown relatively conclusive improvements towards higher and more stable production and we can imagine that it would have only a minimal effect on the environment, as only a minimal amount is captured on the seeds.

It seems that the wider use of nanoparticles is hindered by the reluctance that is due to the sometimes unclear properties of NPs and is also due to people not having entirely clear legislation regarding their use. From the point of view of the local governments, it is therefore, an important step to place the use of nanoparticles in a clear legislative framework. From the research side, the way forward is to offer nanoparticles with an innovative composition, for example, for them to be as similar in their properties as possible to naturally occurring substances. In this regard, bio-nano-fertilizers that are prepared by biosynthesis are a promising substance. This, together with a deeper understanding the natural cycle of nanoparticles, will determine how their application will be further perceived by the public and especially, by crop producers.

## Figures and Tables

**Table 1:** Overview of nanomaterials and their effects on seeds.

Principal Component of Nanoparticle/Size/Other Characters *	Plant Species	Effect on Seedlings	Reference
CuO/6.6 nm /NA	*Lactuca sativa* L.	Lower concentrations (up to 40 μg × ml^−1^) slightly increase plant germination	[132]
ZnO/NA/NA	*Chili pepper*	Significant effect on seed germination growth—higher concentrations support germination, root elongation, length of the aerial part and overall plant growth	[133]
AgNPs/2, 22, 29 nm/spherical	*Brassica oleracea* L.	*Xanthomonas campestris* pv. *campestris* elimination—nanoparticles were more effective than standard hot water treatment	[93]
FeS/6–20 nm/spherical with slight agglomeration	*Oryza sativa* L.	*Fusarium verticillioides* elimination effect on the integrity of the cell membrane of the pathogen, along with adverse effects on the reproduction of the pathogen	[98]
ZnO/35–40 nm/rod morphology; TiO_2_/100 nm/spherical; Ag/85 nm/needle morphology	*Capsicum annum* L.	*Aspergillus flavus, Aspergillus niger, Aspergillus fumigatus* and *Colletotrichum capsici* elimination, improved germination by increasing nitrate reductase, antioxidant activity and reactivity of phytohormones	[99]
Chitosan-guar/<100 nm/spherical in agglomerates	*Oryza sativa* L.	*Pyricularia grisea* and *Xanthomonas oryzae* elimination, support for germination and plant growth—faster germination, greater root growth, significant increase in chlorophyll	[100]
Zn-chitosan/200–300 nm/spherical	*Zea mays* L.	*Curvularia lunata* elimination, positive effect on plant growth—higher percentage of chlorophyll, speed of root and plant growth, earlier maturity, spike length	[101]
Cinnamaldehyde encapsulated in alginate/50–300 nm/mostly spherical	*Pisum sativum* L.	*Pseudomonas syringae* pv. *pisi* elimination, significantly faster seed germination, development of stronger plant parts, longer pods with more seeds	[102]
Pt/3.2 nm/Face-Centered Cubic crystal structure; Ag/3.4 nm/NA; Au/2.6 nm/NA	*Pisum sativum* L.	Inhibition of rhizobial colonization and arbuscular mycorrhizal fungi, decrease in germination, significant increase in yield—number of fruits and seeds, the fatal toxic effect of Pt on *Peperomia pellucida* (L.)	[125]
SiO_2_/NA/NA; Pd/NA/NA; Au/NA/NA; Cu/NA/NA	*Lactuca sativa* L.	The antimicrobial effect on soil microflora, decrease in root length, increase in stem length	[103]
ZnO/NA/NA	*Triticum aestivum* L.	Reducing drought stress—increase in the percentage of chlorophyll; increased content of carotenoids—increased photoprotection of plants	[106]
SiO_2_/NA/NA	*Triticum aestivum* L.	Reducing drought stress—higher number of active reaction centers, high absorbance, trapping, and electron transport	[107]
Cu^0^/30–40 nm/NA	*Zea mays* L.	Mitigation of the physiological effects of drought, chlorophyll and carotenoid content and increased activity of antioxidant enzymes	[105]
Fe_2_O_3_/19–30 nm/spherical	*Citrullus lanatus* (Thunb.)	Influencing the production of phytohormones—increased synthesis of 12-oxo-phytodienoic acid (cis-OPDA) and jasmonic acid	[108]
Mn/50 nm/spherical	*Capsicum anuum* L.	Reduction in the effect of soil salinity—specific redistribution of elements in the plant body, higher roots elongation, regulation of manganese superoxide dismutase production	[110]
Fe_2_O_3_/<50 nm/specific surface area of 180 m^2^/g	*Sorghum bicolor* (L.) Moench	Reduction in the effect of soil salinity—the highest increase in stomatal conductance and transpiration rate, increased chlorophyll *a*, *b*, carotenoids and relative water content	[111]
ZnO/21 nm/crystalline	*Lupinus albus* L.	Reduction in the effect of soil salinity—improving photosynthesis by increasing chlorophyll a, chlorophyll b and carotenoids, increased activity of antioxidant enzymes	[112]
ZnO/25 nm/spherical and hexagonal shapes; Se/10–55 nm/spherical	*Brassica napus* L.	Reduction in the effect of soil salinity—shortening the germination time, increased activity of metabolites, increased activity of antioxidant enzymes	[113]
ZnO/20-30 nm/NA; Fe/50–100 nm/NA	*Triticum aestivum* L.	Reduction in Cd uptake from soil—increased elongation of plants and roots, the significant increase in the dry weight of shoots, roots, cobs and grains, significant influence on photosynthetic parameters such as chlorophyll a, chlorophyll b, carotenoids	[114]
Si/NA/NA	*Triticum aestivum* L.	Reduction in Cd uptake from soil—growth improvement, shoot and root dry weight, shoot length, grain weight and ear length and ear dry weight, improved photosynthesis and increased chlorophyll content, reduction of reactive oxygen species values	[115]
Fe/20–30 nm/NA; Cu/15–30 nm/NA	*Triticum aestivum* L.	Increased germination of three varieties—activated proteins involved in the process of seed germination were detected	[116]
Ag/6–36 nm/spherical and ellipsoidal; Ag/40 nm/spherical	Oryza sativa L.	Improvement germination and starch metabolism of aged rice seeds—acceleration of water intake, increase in α-amylase activity	[85]
	*Vicia faba* L.	Reduction in the genotoxic effects—plumule fresh and dry weight and water content were non-significant, significant root elongation, the significant increase in vitality index	[121]
MgO/12 nm/Face Centered Cubic structure	*Vigna radiata* L.	Increase in germination, % germination and elongation of seedlings and roots, increased chlorophyll content	[122]
Mn/21 nm/Cubic-shaped with hydrophilic character	*Vigna radiata* L.	Positive effect on nitrate intake—increases in both Nitrate reductase and Nitrite reductase activities in root and leaf (testing NPs on mice has not shown a danger of manganism to mammals)	[124]
CuO/8–9 nm/spherical; CuO coated with APTES/10–12 nm/spherical in agglomerates	*Coriandrum sativum* L.	Positive effect on germination with increasing amount of absorbed NPs	[123]
Ag/29 nm/spherical and ellipsoidal	*Citrullus lanatus* (Thunb.)	Increased germination—monitoring of carbohydrate metabolism confirmed the beneficial effect of Nanopriming, increase in the content of photosynthetic pigments, larger stem diameter, longer shoot length, and higher fruit yield, Ag was detected in the seeds of the fruit	[120]
ZnO/13 nm/spherical in agglomerates	*Triticum aestivum* L.	Increased germination and growth—significant increase in the length of roots, shoots and leaves, no significant effect on the number of roots	[130]
Zn-30/30 nm /spherical; Zn-50/50 nm /spherical; Zn-90/90 nm /columnar; Zn-150/150 nm /hexagonal rod-like	*Brassica pekinensis* L.	Germination not affected, significant inhibition of root growth, less inhibition of shoot growth, smaller NPs showed greater phytotoxicity	[131]
Ag_2_S/10–50 nm/spherical in agglomerates; ZnS/5–80 nm/spherical, rod, bean like	*Glycine max* L., *Triticum aestivum* L.	Increased germination but slowing plant growth, longer root and shoot length, longer soaking time and higher concentration caused inhibition of germination and growth	[126]
FeO/20–50 nm/irregular surfaces	*Oryza sativa* L.	Increased germination—faster water absorption, increased % germination, shorter germination time, significant stimulation of α-amylase and antioxidant enzymes	[127]
Fe_2_O_3_/80 nm/irregular	*Triticum aestivum* L.	Increased germination—significantly higher shoot growth, higher chlorophyll formation, nanopriming caused significant accumulation of Fe in harvested seeds	[128]
Fe/SiO/30–40 nm/spherical	*Zea mays* L., *Hordeum vulgare*	Increased germination—faster germination and plant growing	[129]

* if the respective article does not contain the given information, the parameter will be marked as NA (not analyzed).

**Table 2:** Overview of nanomaterials and their effects on seedlings.

Principal Component of Nanoparticle/Size/Other Characters *	Plant Species	Effect on Seedlings	Effect on Plant Physiology	Reference
Ag/15-100 nm/spherical	*Triticum aestivum* L.	Reducing the infestation of seedlings by *Fusarium culmorum*, inhibiting plant growth	Induction of photosynthesis	[135]
Cu/NA/NA; Ag/NA/NA	*Quercus robur* L.	Support of ectomycorrhizal colonization, inhibition of Erysiphe alphitoides	Change of plastids shape and starch content	[138]
Ag/20–100 nm/ spherical	*Gossypium hirsutum* L.	*Xanthomonas axonopodis* pv*. Malvacearum* and *Xanthomonas campestris* pv. *Campestris* elimination	NA, but no phytotoxicity was observed	[109]
Ag-chitosan/≤100 nm/nano composite	*Fragaria* × *ananassa* Duchesne ex Rozier	*Botritis cinerea* elimination	Coated strawberry with nano CTS-Ag had a fresh-like appearance after storage.	[139]
Ag-Si/30 nm/spherical	*Arabidopsis thaliana* L.	*Pseudomonas syringae* pv. *Tomato* elimination	NP regulated the expression of SAR marker genes such as *PR1*, *PR2* and *PR5* in plants	[140]
Ag grown on graphene oxide/± 18 nm ± 5 nm/spherical on the surface of the GO layer	*Lycopersicon esculentum* Mill.	*Xanthomonas perforans* elimination	NA, but no phytotoxicity was observed	[141]
Cu/5–50 nm/spherical in aggregates	*Camellia sinensis* Kuntze	*Poria hypolateritia* elimination	A significant increase in the yield of tea leaves	[147]
Cu, CuO, Cu_2_O Cu/Cu_2_O/11–55 nm/spherical	*Lycopersicon esculentum* Mill.	*Phytophthora infestans* elimination	NA, but no phytotoxicity was observed	[148]
Cu-chitosan/88 nm/spherical	*Eleusine coracana* Gaertn.	*Pyricularia grisea* suppression	CuChNp treatment interferes with the action of endogenous plant hormones and induces changes in the growth profile of treated plants	[149]
Zinkicide SG4/0.2–0.5 nm/ plate-like;Zinkicide SG6/4–6 nm/gel-like structure	*Citrus* × *paradisi* Macfad.	*Xanthomonas citri* subsp. *Citri, Escherichia coli* and *Xanthomonas alfalfae* subsp. *Citrumelonis* elimination	No specific risk of the zinc to plants was observed	[153]
ZnO/25–1500 nm/agglomerated	*Fragaria* × *ananassa* Duchesne ex Rozier	*Botritis cinerea* elimination	Increased flower’s production, reduced growth of runners	[154]
GO/0.76 nm/layer; rGO/1,59 nm/layer	*Oryza sativa* L.	*Xanthomonas oryzae pv. oryzae* elimination	NA	[157]
GO/NA/layer, ZnO/≤ 40 nm/NA	*Daucus carota* L.	*Pectobacterium carotovorum, Xanthomonas campestris pv. carotae, Meloidogyne javanica, Alternaria dauci* and *Fusarium solani* elimination	Significant increase in the content of chlorophyll, carotenoids, proline and overall plant growth	[159]
MgO/20–200 nm/crystalline	*Lycopersicon esculentum* Mill.	*Ralstonia solanacearum* elimination	Resistance induced by NPs by activation of SA-, JA- and ET-signaling pathways and with accumulation of β-1,3-glucanase and tylose	[163]
Al_2_O_3_/100–250 nm/spherical	*Lycopersicon esculentum* Mill.	*Fusarium oxysporium* elimination	Increase of plant height, fresh weight and dry weight	[164]
CH_3_CO-PLA-NS/543 nm/agglomerate; HOOC-PLA-NS/456 nm/agglomerate; H_2_N-PLA-NS/429 nm/agglomerate	*Cucumis sativus* L.	*Streptomyces avermitilis* elimination	The efficient deposition and strong adhesion of pesticides on the leaf surface	[10]
ZnO/15–137 nm/mostly spherical	*Coffea arabica* L.	Increased growth and biomass production	Increase in fresh weight of roots and leaves	[175]
ZnO/1.2–6.8 nm/oblate spherical and hexagonal	*Cyamopsis tetragonoloba* L.	Increased growth and biomass production	Improvement in shoot length, root length, root area, chlorophyll content, total soluble leaf protein, rhizospheric microbial population, acid phosphatase, alkaline phosphatase and phytase	[176]
ZnO/16–30 nm/spherical	*Cicer arietinum* L.	Effect on biomass production	Improving total dry matter accumulation	[24]
Zn/NA/NA; Fe/NA/NA; Mn/NA/NA	*Phaseolus vulgaris* L.	Increased growth and biomass production	NA	[177]
TiO_2_/25 nm/; ZnO/28 nm/	*Lycopersicon esculentum* Mill.	Increased growth and biomass production	TiO_2_ and ZnO (>250 mg.kg^−1^) caused root reduction	[178]
Ag/35–40 nm/NA	*Triticum aestivum* L.,*Vigna sinensis* (L.) Endl. ex Hassk., *Brassica juncea* L.	Increased growth and biomass production	Increased root nodulation (*Vigna*)	[179]
chitosan-NPK/26–30 nm/NA	*Triticum aestivum* L.	Increased growth and biomass production, higher and earlier yield	NA	[180]
GO/300 nm–5µm/multilayers nanolayers	*Zea mays* L.	Effect on biomass production; without effect on bacteria	A significant negative effect on root growth was observed in watered plants	[181]
Fe_2_O_3_/10–50 nm/spherical	*Arachis hypogaea* L.	Increasing plant height and iron content in plants	Regulation of phytohormone content and antioxidant enzymatic activity	[182]
Fe/NA/NA; Zn/NA/NA	*Zea mays* L.	Increased growth and biomass production	Increasing the phosphorus, leaf chlorophyll, crude protein andsoluble carbohydrate concentration compared to chemicalforms	[183]
TiO_2_/30 nm/oblate spherical; ZnO/20 nm/spherical; Fe_2_O_3_/80 nm/spherical	*Hordeum vulgare* L.	Increase in growth properties and greater grain production	The chlorophyll content was significantly increased—increased photosynthesis—due to the increased formation of assimilates, the number and weight of seeds increased	[184]
TiO_2_/NA/anatase and rutile structures	*Oryza sativa L.*	Significant reduction in intake of As plant roots	Prevention of penetration of As into the root, restriction of movement and isolation in vacuoles in the root cells	[136]
CeO_2_ + polyvinilpyrolidon/30–50 nm/spherical	synthetic root environment	Suppression of Cd and As uptake from soil solution in the presence of synthetic root exudates (SRE)	NA	[185]
Si/NA/NA; TiO_2_/NA/NA	*Oryza sativa* L.	Increase in biomass production and a lower accumulation of cadmium in plants	Increase in dry weight, increase in chlorophyll content—acceleration of photosynthesis, stomatal conductance, transpiration rate and activity of anti-oxidative enzymes	[186]
SiO_2_/NA/NA	*Larix* sp.	Increased growth	Increase in chlorophyll content	[187]
SiO_2_/10–15 nm/amorphous	*Prunus mahaleb* L., *Crataegus azarolus* L.	Increasing the growth and efficiency of photosynthesis	Slight improvement in leaf physiological performance and root elongation	[188]
Cu encapsulated in a carbon shell/50 nm/spherical	*Taxodium distichum*	Wood protection against *Trametes versicolor* L., *Ophiostoma minus* Syd and P.Syd and *Coptotermes formosanus* Shiraki	Facilitate copper transport by plant roots and increased Cu uptake	[189]
CeO_2_ NPs/4 nm/specific surface area; Fe_2_O_3_ NPs/6 nm/hematite phase;SnO_2_ NPs/6 nm/NA; TiO_2_ NPs/6 nm/anatase phaseSiO_2_ NPs/10 nm/specific surface area	*Lactuca sativa L. var. foliosa*	An effect on the metabolism and modification of the physiological functions of the plants was observed	Influence on the amount and activity of APX, GPOX, CAT, GSH, carotenoids, chlorophyll A + B and on the amount of dry matter	[12]

* if the respective article does not contain the given information, the parameter will be marked as NA (not analyzed).

## Data Availability

Not applicable.
